# Level and determinants of diabetes knowledge in patients with diabetes in Zimbabwe: a cross-sectional study

**Published:** 2012-12-12

**Authors:** Esther Mufunda, Kerstin Wikby, Albin Björn, Katarina Hjelm

**Affiliations:** 1 School of Health and Caring Sciences, Linnaeus University, Växjö, Sweden; 2 Department of Health Sciences, Zimbabwe Open University, Harare, Zimbabwe

**Keywords:** Diabetes mellitus, diabetes knowledge, determinants, diabetes education, descriptive cross-sectional study, self-report questionnaire, Zimbabwe

## Abstract

**Introduction:**

A previous study of beliefs about health and illness in Zimbabweans with diabetes mellitus indicated limited knowledge about diabetes and the body, affecting self-care and health-care seeking behaviour. The aim of this study was to assess the level of diabetes knowledge in Zimbabwean adults with diabetes mellitus, to determine the main gaps in knowledge and identify the socio-demographic and diabetes-related determinants that predict diabetes awareness and self-care practices.

**Methods:**

A cross-sectional descriptive study was performed using a standardized self-report Diabetes Knowledge Test questionnaire (DKT) of 58 respondents, 32 women and 26 men. Results were analysed with descriptive and analytic statistical methods.

**Results:**

The majority of the respondents scored average knowledge on all three sub-scales: general knowledge, insulin use and total knowledge, with an overall score of 63.1± 14, 2%. Major knowledge gaps were in areas related to diet, insulin use and glycaemic control. No significant differences in mean scores were detected in the diabetes knowledge sub-scales when comparisons were made of mean knowledge scores in relation to socio-demographic and diabetes-related characteristics. However, diabetes-related complications were significantly associated with lower total and general diabetes knowledge, and female gender was an independent determinant of low general knowledge.

**Conclusion:**

Knowledge gaps were evident in areas regarding insulin use, diet and glycaemic control. Low diabetes knowledge was associated with female gender and could be a risk factor for development of diabetes-related complications. Knowledge gaps need to be addressed in diabetes education to prevent development of diabetes-related complications.

## Introduction

Diabetes mellitus affects millions of people worldwide and its related complications continue to be of great concern. The pandemic is rapidly spreading in developing countries and particularly affecting poor populations in Sub-Saharan Africa [1, 2].

Globally, communicable diseases are slowly being replaced by non-communicable diseases (NCDs), diabetes mellitus being amongst them, associated with increased modernization, wealth and prosperity [3]. In developing countries, the incidence of NCDs correlates with the degree of modernization and lifestyle changes, thus placing a double burden of diseases on people in the Sub-Saharan countries [4].

Diabetes mellitus is ranked fifth amongst the NCDs recorded in most Zimbabwean public hospitals, with a prevalence rate of 10% in the ≥ 25 age group [5]. A previous study of beliefs about health and illness in Zimbabweans diagnosed with diabetes mellitus indicated limited knowledge about both diabetes and the body [6]. It was further reported that the limited knowledge about DM affected self-care and health-seeking behaviours amongst Zimbabwean males and females with DM [7], although this was less marked in comparison with findings from a related Ugandan study [8]. From both these studies, gender seemed to influence the risk awareness of the disease, with females thus being more information-seeking and active in self-care. However, irrespective of gender, limited diabetes knowledge and self-care was indicated [6–8].

With a few exceptions [9–11], no other studies investigating knowledge of diabetes and knowledge gaps have been found in African populations. All previous studies concluded limited knowledge about diabetes, the management and patient self-care. However, the findings were not generalizable as the studies used either a qualitative [9] or a quantitative study design [10, 11], were based on a convenient sample of limited size [11] and had instruments developed from literature review and in relation to the studied area [10, 11]. Studies performed in countries outside Africa used the Diabetes Knowledge Test (DKT) [12, 13], and even in these studies inadequate knowledge was evident.

With the increased prevalence of diabetes mellitus and ever decreasing health care resources especially in developing countries, the need for self-care management becomes paramount. It has been reported that patients with diabetes often lack sufficient knowledge about their disease and thus frequently have poor self-care management [14]. The outcome of diabetes depends mainly on the patient's self-management [12, 15] including health-related behaviour which is determined by individual beliefs about health and illness, based on his or her knowledge [16, 17].

Health education by health care staff and employing new research findings and useful strategies can reduce the burden of the disease [16]. Nurses play an important role in fighting the pandemic and the burden of it by working with health-promoting education [16], particularly to enable the patients to take responsibility for their lives and help them feel safer in making their own decisions and to improve their knowledge and attitudes towards their health [18].

### The study

The aim of the study was to assess the level of diabetes knowledge in Zimbabwean adults with diabetes mellitus, to determine the main gaps in knowledge and identify the socio-demographic and diabetes-related determinants that predict diabetes awareness and self-care practices.

## Methods

### Design

A cross-sectional descriptive study was conducted using a standardized self-report questionnaire, Diabetes Knowledge Test (DKT), to investigate knowledge about diabetes. The design enabled the investigator to gather information on the variable of interest in persons with diabetes mellitus and to make inferences about possible relationships between diabetes knowledge and the persons’ socio-demographic characteristics [19].

### Participants

The sample consisted of 58 adult respondents, 32 women and 26 men, with diabetes mellitus obtained through convenience sampling.The inclusion criteria were: diagnosis with diabetes mellitus for ≥ one year, mentally sound to give informed consent and be conversant with either English or Shona (two of the three official languages in Zimbabwe [5]

### Data collection

A standardized self-report questionnaire (DKT) was used to collect data on the respondents’ knowledge about diabetes, its treatment, their understanding, socio-demographic and diabetes-related background data. The participants were attending meetings at the Zimbabwe Diabetes Association. This is anaffiliated non-governmental patient organization that helps in giving both material and information support as part of continued self-management to persons with diabetes who are usually referred from the hospital-based diabetes clinics. One reason for attending the sessions was availability and lower prices of insulin than in public health care pharmacies. Another reason for attending was sharing of disease experiences and getting information about diabetes and the management based on patients’ desires on information for example about diet, physical exercises, medication, self-care etc. Meetings are held monthly for about 2 hours.

A registered nurse (first author) distributed the questionnaires when the respondents met. If needed she clarified and answered questions from the participants during the completion of the questionnaires. The total time required to complete the questionnaire was about an hour.

### Data collection instrument

Data were collected using a standardized self-report questionnaire including the Diabetes Knowledge Test (DKT) of the University of Michigan Diabetes Research and Training Centre and socio-demographic and diabetes-related background data.

The DKT has been shown to be a reliable and valid instrument [20] consisting of 23 questions testing the patients’ general understanding of diabetes with respect to diet, blood glucose monitoring, foot care, diabetes complications, proper insulin usage, adverse effects of insulin and factors that influence blood glucose levels. It has two parts, a 14-item general knowledge test and a 9-item insulin-use subscale.Addition of the two gives a score of the total knowledge about diabetes.

The socio-demographic and diabetes-related background data included sex, age, educational level, marital status, employment status, duration of diabetes mellitus, type of diabetes treatment, diabetes-related complications, duration of attending follow-ups and attendance of diabetes classes.

The original questionnaire in English was translated into Shona by a bilingual Shona-speaking senior nurse (first author), then translated back into English by an independent Shona-speaking senior diabetes educator. Comparison of the two versions for content and meaning was done and agreed upon by the principal investigator (first author) and the co-authors. The questionnaire was then pre-tested on four persons who met the set inclusion criteria (but were not included in the final sample) before being used in order to test the questions and to estimate how long it took to complete the questionnaire.

### Ethical considerations

The study was approved by the Diabetes Association of Zimbabwe and the Medical Research Council of Zimbabwe. It was done in accordance with the Helsinki Declaration [21] and with written informed consent obtained from the respondents.

### Data analysis

Data were checked regarding underlying distributional assumptions, described and analysed accordingly. For normally distributed variables, Student's t-test was used for group comparisons and one-way analysis of variance (ANOVA) used to compare > 2 groups. For the non-normally distributed variables, the non-parametric Mann-Whitney U-test was performed to compare two groups and the Kruskal-Wallis test was used to compare more than two groups. Pearson's chi-squared test was used to ascertain any association between two qualitative variables.

Three multiple logistic regression analyses were performed to identify any independent association between the participants’ socio-demographic and diabetes-related characteristics and low knowledge levels (general knowledge, insulin use knowledge and total knowledge). As dependent variables the third of the participants with the lowest knowledge level in the respective subscale were chosen. The analysis was performed in steps, with all independent variables included in the first step. Subsequently, step by step, the variable in the previous step with the highest p-value was excluded from the model. A p-value < 0.05 was considered statistically significant [19].

The total knowledge score was determined by awarding one point for each correct answer and a zero for a wrong answer or no response. The total knowledge score ranged from 0-23 and was categorized as follows: < 11= poor knowledge, 11–17= average knowledge and >17 = good knowledge. The general knowledge score was categorized as: < 7= poor, 7–11= average, > 11 = good and insulin use knowledge was categorized as follows: < 5= poor, 5–7 =average and > 7 = good [12]. Knowledge gaps were then identified by recording questions in the DKT incorrectly answered by more than 50% of the respondents. Data were analysed using SPSS version 19 (SPSS Inc, IL, USA).

## Results

### Respondents’ socio-demographic and diabetes-related data

Table 1 summarizes the socio-demographic characteristics of the respondents. A total of 61 questionnaires were distributed and 58 respondents completed the questionnaires fully while three did not return the questionnaires, giving a response rate of 95%. Of the 58 respondents, 32 were females (55%) aged 19-70 years (median 35 years) while 26 were males (45%) aged 20-72 years (median 33 years). About half of the respondents were married, and had education above secondary school level, while almost two thirds were unemployed. More than half were treated with insulin and most had had DM for 4-9 years (median 8 years), with about one third reporting diabetes-related complications. The majority of respondents stated that they attended diabetes meetings.

**Table 1:** Socio-demographic and diabetes-related characteristics of the respondents

Variable	Frequency, n (%)
**Gender**	
Male	26 (45)
Female	32 (55)
**Marital status**	
Unmarried	17 (29)
Married/cohabitant	29 (50)
Divorced/separated	5 (9)
Widow/widower	7 (12)
**Educational level**	
Primary school	5 (9)
Secondary school	23 (40)
Tertiary/college	18 (31)
University < 2 years	1 (2)
University> 3 years	11 (19)
**Employment status**	
Unemployed	34 (59)
Gainfully employed	18 (31)
Sick leave	2 (3)
Retired	4 (7)
**Duration of diabetes in years**	
≤3	11 (19)
4 - 9	22 (38)
10 - 15	11 (19)
≥ 16	14 (24)
**Diabetes treatment regimen**	
Diet	4 (7)
Oral agents	20 (35)
Insulin	32 (55)
Combination of insulin and oral agents	2 (3)
**Diabetes-related complications**	16 (28)
**Attendance at Diabetes class**	41 (71)
**Duration of Diabetes class attendance in years**	
≤3	16 (28)
4 - 9	25 (43)
≥ 10	15 (26)
Missing	2 (3)

### Level of diabetes knowledge

Overall 63.1 ± 14.2% of the respondents correctly answered the items on the total DKT while 63.8 ± 15.2% had comparable scores on the general knowledge subscale and 62.1 ± 19.7% on the insulin use knowledge subscale (data not shown). No gender differences were found (Table 2). A fifth (20.7%) of the respondents scored ‘good’ on the total DKT, 10.3% scored ‘good’ in general knowledge and 15.5% in insulin use. The majority of respondents scored average knowledge on all three knowledge sub-scales (Table 3).

**Table 2:** Comparison between males’ and females’ knowledge about diabetes

	Males	Females	
	Mean (SD)%	Mean (SD)%	P value
Total knowledge	65.2 (14.2)	61.4 (14.1)	0.875
General knowledge	66.2 (13.5)	61.8 (16.4)	0.279
Insulin use knowledge	64.5 (20.1)	60.1 (19.5)	0.397

**Table 3:** Respondents’ knowledge about diabetes

Category scores	Frequency (n)%	Description
**Total knowledge (out of 23)**		
< 11	7 (12.1)	Poor
11–17	39 (67.2)	Average
≥18	12 (20.7)	Good
**General knowledge (out of 14)**		
< 7	17 (29.3)	Poor
7–11	35 (60.3)	Average
≥ 12	6 (10.3)	Good
**Insulin use knowledge (out of 9)**		
< 5	14 (24.1)	Poor
5–7	35 (60.3)	Average
≥8	9 (15.5)	Good

Knowledge deficit (incorrect answers above 50%) was noted in six questions; in descending order these were related to the definition of a ‘free food’ (75.9%), the purpose of testing HbA1C (67.2%), cause of an insulin reaction (67.2%), food that should not be used to treat low blood glucose (58.6%), signs of ketoacidosis (58.6%) and what to do when one starts to have an insulin reaction (51.7%) (Table 4).

**Table 4:** Questions most commonly answered either incorrectly or not at all

Answer (*correct answer in italics*)	Incorrect (%)	No answer (%)
Which of the following is a ‘free food’? ‡ any unsweetened food ‡ any dietetic food ‡ any food that says ‘sugar free’ on the label ‡ *any food that has less than 20 calories per serving*	75.9	0
Glycosylated haemoglobin (haemoglobin A1) is a test that is a measure of your average blood glucose level for the past: ‡ day ‡ week ‡ *6–10 weeks* ‡ 6 months	67.2	0
Which one of the following will most likely cause an insulin reaction? ‡ *heavy exercise* ‡ infection ‡ overeating ‡ not taking your insulin	67.2	0
Which should not be used to treat low blood glucose? ‡ 3 hard candies ‡ ½ cup orange juice ‡*1 cup diet soft drink* ‡ 1 cup skim milk	58.6	0
Signs of ketoacidosis include: ‡ shakiness ‡ sweating ‡ *vomiting* ‡ low blood glucose	58.6	2
If you are beginning to have an insulin reaction, you should: ‡ exercise ‡ lie down and rest ‡ *drink some juice* ‡ take regular insulin	51.7	0

Comparisons of the mean knowledge scores in relation to the participants’ socio-demographic characteristics and diabetes-related data were made (Table 5). No significant differences in mean scores were detected either in general knowledge, insulin use knowledge or total knowledge about diabetes, with one exception. Reporting diabetes-related complications was significantly associated with lower total and general diabetes knowledge (p 0.028 vs 0.008). The mean scores in insulin use knowledge were similar for those who attended and those who did not attend regular diabetes meetings.

**Table 5:** Comparison of mean knowledge scores according to respondents’ socio-demographic and diabetes-related data

Variable	General knowledge score Mean ± SD	P-value	Insulin use knowledge score Mean ± SD	P-value	Total knowledge score Mean ± SD	P-value
**Gender**		**0.102**		**0.220**		**0.087**
Male	9.4 ± 1.8		5.8 ± 1.8		15.2 ± 3.2	
Female	8.6 ± 2.3		5.4 ± 1.7		14.0 ± 3.2	
**Marital status**		**1.00**		**0.559**		**0.556**
Married/cohabitant	8.9 ± 2.3		5.4 ± 1.6		14.4 ± 3.4	
Unmarried	8.9 ± 2.0		5.7 ± 1.9		14.7 ±3. 2	
**Educational level**		**0.483**		**0.311**		**0.288**
Low educational level (primary/ secondary school)	8.4 ± 1.9		5.6 ± 1.6		14.0 ± 2.8	
High educational level (college/ university)	9.4 ± 2.2		5.5 ± 1.9		15.0 ± 3.6	
**Employment**		**0.927**		**0.330**		**0.152**
Unemployed	8.9 ± 2.1		5.7 ± 1.7		14.6 ± 3.0	
Gainfully employed	9.0 ± 2.3		5.4 ± 2.0		14.4 ± 3.7	
**Diabetes treatment regimen**		**0.875**		**0.334**		**0.957**
Diet only	9.5 ± 1.3		4.3 ± 1.0		13.8 ± 1.9	
Oral agents	9.1 ± 1.9		5.8 ± 1.5		14.8 ± 3.0	
Insulin	8.8 ± 2.4		5.7 ± 1.9		14.4 ± 3.5	
Combination (insulin and oral) agents	9.5 ± 2.1		5.0 ± 4.2		14.5 ± 6.4	
**Diabetes-related complications**		0.008*		**0.244**		0.028*
Yes	7.8 ± 2.2		5.1 ± 1.9		12.8 ± 3.5	
No	9.4 ± 1.9		5.8 ± 1.7		15.2 ± 3.0	
**Attended diabetes sessions**		**0.836**		**0.945**		**0.803**
Yes	9.0 ± 2.3		5.6 ± 1.7		14.6 ± 3.2	
No	8.8 ± 1.8		5.6 ± 2.1		14.4 ± 3.4	

* p < 0.05

### Independent associations of knowledge with the socio-demographic variables

Multiple logistic-regression analyses were used to estimate the independent associations of between poor knowledge and socio-demographic variables and diabetes-related characteristics. Female gender was an independent determinant of low general knowledge about diabetes (OR= 3.5; 95% CI 1.2–10.6, p= 0.028, b=1.250), while no significant associations were found between insulin use knowledge, total knowledge and the respondents’ demographic and diabetes-related characteristics (data not shown).

## Discussion

As far as we know, this is the first study investigating diabetes knowledge using a validated instrument in an African population. Overall, 63.1± 14.2% of the respondents correctly answered the DKT questionnaire with the majority scoring average knowledge in all three knowledge sub-scales: general knowledge, insulin use and total knowledge. Major knowledge gaps were noted in six questions related to insulin use, glycaemic control and diet as noted by scores below 50%. Reporting diabetes-related complications was significantly related to low total and general knowledge about diabetes, and female gender was found to be an independent determinant of low general knowledge of diabetes.

Diabetes outcome depends mainly on the patient’ sound knowledge of self-care that is dependent upon their knowledge of the disease [16, 17, 24], including health-related behaviour and care-seeking [25, 26] which are guided and determined by individually and culturally defined beliefs about health, illness and health-care [6, 27]. It is reported that patients with low diabetes knowledge levels are least likely to comply with diabetes management and instructions from health-care professionals [28]. In this study of adults with diabetes mellitus and having different types of treatment indicating different types of diabetes (Type 1 and 2 diabetes), the results show that most of them had average knowledge about diabetes. No significant gender differences were noted in knowledge scores. Although there are no completely comparable studies on African populations, the present study results are similar to those found in three other studies from both developing [12] and developed countries [13, 22] using the DKT questionnaire. Findings from these studies reported the same diabetes knowledge levels in patients with Type 2 [12, 22] and those with Type 1 or Type 2 diabetes [13].

The major knowledge gaps reported in this study in the areas related to diet, insulin use and glycaemic control are almost consistent with previous studies which also used the DKT, and respondents scored less than 50% in the same questions [12, 22]. In our study, it might appear that for some of the respondents, the main reason for attending the Diabetic Association meetings was not just for educational purposes but other reasons such as getting cheaper insulin, blood sugar testing, sharing of disease experiences etc., and hence they did not seem to benefit from the health-education sessions. However, this finding should be a major impetus to develop the content of diabetes education, as almost two thirds of the respondents were on insulin treatment and at greater risk of developing insulin-related complications. A previous study of persons with Type 2 diabetes, who were following a diabetes educational programme, attributed the poor knowledge scores to possible poor understanding of terms such as ketoacidosis, insulin reaction, free food, HaemoglobinA1C or carbohydrates [22], but in this study it was difficult to ascertain this as all the respondents completed the questionnaires in full. It is therefore important to identify knowledge gaps to allow for better focused educational initiatives that address specific diabetes self-care practices [13, 23] and barriers to ongoing educational programmes.

Previous studies performed in African populations reported females being more active in self-care and information seeking than males [6–8], but in this study being female was an important determinant of low general diabetes knowledge. This finding might seem contradictory, but it could be a major indication of lack of access to information about DM irrespective of a more active information-seeking behaviour. Previous studies have however shown contradictory results about the relationship between gender and level of knowledge [12, 22]. It is therefore important that nurses and other health care practitioners realize the importance of gender-sensitive diabetes self-management education (DSME) based on individual needs, which is the cornerstone of care for patients with diabetes as it improves patient outcomes [12] and reduces the global burden of diabetes [29].

Diabetes-related complications was an important factor for low diabetes knowledge (general and total knowledge), which could be an indication of low knowledge as a risk factor for developing diabetes-related complications. This finding could further support the reported knowledge gaps in glycaemic control, diet and insulin use, another implication for the importance of addressing these areas through diabetes education programmes and periodically re-assessing individual patient's educational needs and barriers to ongoing diabetes education based on individual beliefs about health and illness [6–8, 17].

The main strength of this study was the use of a validated DKT questionnaire [20] and similar results to previous studies from both developing [12] and industrialized countries [13, 22]. The major limitations of the study concern the sample. There is a possibility of selection bias since the study population was limited and recruited from a patient association offering different activities such as diabetes education, giving patients an enabling environment to share disease experiences and access to low-cost insulin. It would then be assumed that the respondents would have more knowledge than the general population. The findings could be different if a general population had been used. Another selection bias could be that the studied population has a higher educational background (about half had educational levels above secondary school) than in the previous study from a developing country [12] where most were illiterate. However, the present sample is similar in characteristics to the general population concerning high unemployment rate and gender distribution. The low median age (35 vs 33 years) can be explained as a consequence of that the life expectancy in Zimbabwe in 2010 was reported as 49.9 years [30].

A study sample in a general population of persons with diabetes might have given different results and possibly even lower knowledge levels.

## Conclusion

This study has some areas of knowledge deficit with regard to diet, glycaemic control and insulin use. Low diabetes knowledge was significantly associated with female gender and could be a risk factor for development of diabetes-related complications. The areas of knowledge gaps need to be addressed in diabetes education as they might influence the development of diabetes-related complications. However, the limited sample size restricts the possibility to draw conclusions from our results to the general diabetes population in Zimbabwe why further studies of a representative sample are needed.
